# Nuclear-Cytoplasmic Partitioning of Cucumber Mosaic Virus Protein 2b Determines the Balance between Its Roles as a Virulence Determinant and an RNA-Silencing Suppressor

**DOI:** 10.1128/JVI.00284-14

**Published:** 2014-05

**Authors:** Zhiyou Du, Aizhong Chen, Wenhu Chen, Qiansheng Liao, Hengmu Zhang, Yiming Bao, Marilyn J. Roossinck, John P. Carr

**Affiliations:** a College of Life Sciences, Zhejiang Sci-Tech University, Hangzhou, China; b Department of Plant Sciences, University of Cambridge, Cambridge, United Kingdom; c State Key Laboratory Breeding Base for Zhejiang Sustainable Pest and Disease Control, Zhejiang Academy of Agricultural Sciences, Hangzhou, China; d Center for Infectious Disease Dynamics, Pennsylvania State University, University Park, Pennsylvania, USA

## Abstract

The Cucumber Mosaic Virus (CMV) 2b protein is an RNA-silencing suppressor that plays roles in CMV accumulation and virulence. The 2b proteins of subgroup IA CMV strains partition between the nucleus and cytoplasm, but the biological significance of this is uncertain. We fused an additional nuclear localization signal (NLS) to the 2b protein of subgroup IA strain Fny-CMV to create 2b-NLS and tested its effects on subcellular distribution, silencing, and virulence. The additional NLS enhanced 2b protein nuclear and nucleolar accumulation, but nuclear and nucleolar enrichment correlated with markedly diminished silencing suppressor activity in patch assays and abolished 2b protein-mediated disruption of microRNA activity in transgenic Arabidopsis. Nucleus/nucleolus-localized 2b protein possesses at least some ability to inhibit antiviral silencing, but this was not sufficient to prevent recovery from disease in younger, developing leaves in Arabidopsis. However, enhanced nuclear and nucleolar accumulation of 2b increased virulence and accelerated symptom appearance in older leaves. Experiments with Arabidopsis lines carrying mutant *Dicer-like* alleles demonstrated that compromised suppressor activity explained the diminished ability of 2b-NLS to enhance virus accumulation. Remarkably, the increased virulence that 2b-NLS engendered was unrelated to effects on microRNA- or short interfering RNA-regulated host functions. Thus, although nucleus- and nucleolus-localized 2b protein is less efficient at silencing suppression than cytoplasm-localized 2b, it enhances CMV virulence. We propose that partitioning of the 2b protein between the cytoplasmic and nuclear/nucleolar compartments allows CMV to regulate the balance between virus accumulation and damage to the host, presumably to maximize the benefit for the virus.

**IMPORTANCE** In this work, the main finding is that nucleus/nucleolus-localized 2b protein is strongly associated with CMV virulence, which is independent of its effect on small RNA pathways. Moreover, this work supports the contention that the silencing suppressor activity of CMV 2b protein is predominantly exerted by that portion of the 2b protein residing in the cytoplasm. Thus, we propose that partitioning of the 2b protein between the cytoplasmic and nuclear/nucleolar compartments allows CMV to regulate the balance between virus accumulation and damage to the host, presumably to maximize the benefit for the virus.

## INTRODUCTION

In plants, viral disease symptoms result from complex and incompletely understood interactions between viral gene products and the host. Among other things, virus infection triggers short interfering RNA (siRNA)-mediated antiviral defense (RNA silencing) to modulate or inhibit virus infection by targeted destruction of viral RNA molecules (1, 2). To counter this antiviral defense, most plant viruses identified to date encode viral suppressors of RNA silencing (VSRs), which inhibit or inactivate various components of the RNA-silencing pathways (3, 4). Additionally, many VSR molecules play important roles in viral symptom induction (5).

Cucumber Mosaic Virus (CMV) is an economically important pathogen that infects more than 1,200 plant species (6). Numerous CMV strains and isolates have been characterized and can be divided into three subgroups, IA, IB, and II (7). The CMV 2b protein was suggested to be a pathogenicity determinant by initial findings using the subgroup IA strain Fny and the subgroup II strain Q (8, 9). Mutations that prevented 2b translation or deleted the *2b* gene produced viral variants (CMVΔ2b mutants) that induced mild symptoms or no symptoms on a number of solanaceous hosts (8, 9). Subsequently, studies using silencing-compromised Arabidopsis thaliana plants harboring mutant alleles for Dicer-Like (DCL) endoribonucleases that were infected with Q-CMVΔ2b or Fny-CMVΔ2b showed that 2b proteins could also play an indirect role in pathogenicity by inhibiting virus-derived siRNA-mediated antiviral defense, thereby promoting the accumulation of other CMV gene products with virulence functions (10–12).

The CMV 2b protein was one of the first VSRs to be discovered, and it inhibits initiation of RNA silencing in newly emerging tissues (13), probably by blocking systemic propagation of silencing signals identified as small RNA (sRNA) duplexes (14–16). From a study of three VSRs, it was concluded that binding of sRNA duplexes is a mechanism used by many VSRs to inhibit RNA silencing (17). There is now substantial evidence that the 2b protein binds double-stranded microRNA (ds-miRNA) and various double-stranded siRNAs *in vivo* and *in vitro* and that sRNA sequestration is the predominant mechanism by which the 2b protein disrupts silencing pathways (18–22). However, 2b proteins can also interact with various host proteins, including Argonautes (AGO) 1 and 4 (18–23) and catalase 3 (24), and these interactions are thought to contribute in various ways to viral pathogenicity. Experiments with transgenic Arabidopsis plants showed that constitutive expression of the *2b* gene from the subgroup II strain Q or LS had little effect on miRNA-regulated plant development but that transgenic expression of the *2b* gene from subgroup IA strain Fny impaired miRNA regulation of gene expression, leading to developmental defects, concomitant with increased steady-state accumulation of mature miRNA (guide strand) and star miRNA (passenger strand) (23, 25, 26). It was concluded that the effects of the 2b protein on miRNAs explains the induction of severe symptoms by Fny-CMV (23, 26). The failure of LS2b or Q2b to disrupt miRNA functions was suggested to be due either to instability *in planta* (23) or lack of a domain required for interference with miRNA function (26).

Cell fractionation experiments showed that the Fny2b protein was enriched in nucleus- and cytoskeleton-associated insoluble fractions extracted from virus-infected plants (27). Consistent with this, transient expression of green fluorescent protein (GFP) or β-glucuronidase (GUS) fusions with Fny2b or SD2b (from another subgroup IA strain, SD-CMV) in onion or tobacco cells showed that this VSR accumulates not only in the nucleus but also in the cytoplasm (18, 19, 28). Nuclear targeting of 2b proteins from subgroup IA strains is governed by two nuclear localization signals, NLS1 and NLS2 (18, 19, 28). In contrast, GFP or GUS fusions with Q2b accumulate predominantly in the nucleus, with localization determined by a single NLS (29). Additionally, 2b proteins from subgroup IA strains are present in nucleoli (18, 19). Early studies using CMV2b NLS mutants indicated that nuclear enrichment was required for RNA silencing suppression and was associated with CMV pathogenicity (11, 28, 29). Recently, it was shown that the NLS sequence in the Fny2b protein is coincident with the domain required for sRNA binding (19). To discern the relative importance of nuclear localization versus sRNA binding for CMV 2b protein-mediated suppression of RNA silencing, González and colleagues inhibited sustained nuclear accumulation of GFP-Fny2b by fusing a nuclear export signal (NES) to its N terminus (20). They found that whereas sRNA binding activity is required for VSR activity, nuclear localization is dispensable for this function. We wondered what biological significance nucleus-localized Fny2b may have and if other cellular compartments are required for 2b protein-mediated RNA silencing suppression or symptom induction. To do this, we exaggerated the accumulation of the 2b protein from subgroup IA strain Fny in the nucleus relative to the cytoplasm by translational fusion with an additional (third) NLS sequence and tested the effect of this on VSR activity and viral pathogenicity.

## MATERIALS AND METHODS

### Plant materials.

Arabidopsis (Arabidopsis thaliana Heyn. ecotype Col-0) wild-type and mutant plants were grown under an 8-h photoperiod and a light intensity of 150 to 200 μE · m^−2^ · s^−1^ at 22°C. Arabidopsis mutant lines *dcl1-9*, *dcl3-1*, and *dcl2-1/dcl4-2* were described previously (30–32). Nicotiana benthamiana, Nicotiana glutinosa, and Nicotiana tabacum plants were grown under a 16-h photoperiod with a light intensity of 150 to 200 μE · m^−2^ · s^−1^ at 25°C.

### DNA constructs.

The binary vector p35S:GFP derived from the plasmid pBI121, which carries a modified *GFP* (mgfp5) sequence under the control of the constitutive Cauliflower mosaic virus 35S promoter was described previously (13). To express enhanced GFP (EGFP)-tagged Fny2b or LS2b protein in plants, we constructed two additional pBI121-derived plasmids, pBI121-EGFP-Fny2b and pBI121-EGFP-LS2b. Briefly, a DNA construct encoding a protein in which EGFP was fused to the N terminus of Fny2b or LS2b was created by overlapping PCR using partially complementary primers. The PCR product was cloned into the pRTL2 plasmid after digestion with restriction endonuclease enzymes NdeI and SacI, to generate plasmids pG:Fny2b and pG:LS2b. Both plasmids were digested using HindIII and SacI and subcloned into pBI121 to generate pBI121-EGFP-Fny2b and pBI121-EGFP-LS2b. To construct pBI121-EGFP, a DNA fragment was amplified by PCR using pBI121-EGFP-Fny2b as the template with primers 35S-XhoI/GFP-R-SacI. The resultant PCR product was digested with XhoI and SacI and inserted into the pBI121-EGFP-Fny2b predigested with XhoI and SacI, giving pBI121-EGFP.

For generation of pBI121-EGFP-Fny2b derivatives, site-directed mutations were introduced into Fny2b by PCR using mutagenic oligonucleotides (F2b-K23R-R, F2b-Q24R-R, F2b-E35A-F, F2b-K23R-Q24R, F2b-NLS-SacI-R, and F2b-6His-Sac-R) to produce DNA fragments GFP-Fny2bK23R, GFP-Fny2bQ24R, GFP-Fny2bE35A, GFP-Fny2bRRA, GFP-Fny2bNLS, and GFP-Fny2b6His. These DNA fragments were digested with XhoI and SacI and subsequently inserted into the predigested pBI121-EGFP-Fny2b plasmid to generate constructs pBI121-EGFP-Fny2bK23R, pBI121-EGFP-Fny2bQ24R, pBI121-EGFP-Fny2bE35A, pBI121-EGFP-Fny2bRRA, pBI121-EGFP-Fny2bNLS, and pBI121-EGFP-Fny2b6His. Prior to transformation of these constructs into Agrobacterium tumefaciens GV3101, all constructs were authenticated by DNA sequencing.

Infectious clones (pFny109, pFny209, and pFny309) of Fny-CMV have been described previously (33). The plasmid pFny209Δ2bpro, a derivative of pFny209 in which the 2b open reading frame was rendered untranslatable, has been described previously (34). Infectious clones pFny209-2bNLS and pFny209-2b6His, in which the 2b protein is fused with an NLS motif (KRRRRR) or a hexahistidine sequence at its C terminus, were generated as previously described (34). Briefly, the forward primer C2F1856 and mutagenic primers (either Fny2b-NLS-R or Fny2b-6His-R) were used to amplify DNA fragment I using pFny209 as the template, and the reverse primers CMV123R and Fny2b-NLS-F or Fny2b-6His-F were used to amplify DNA fragment II using pFny209 as the template. Fragments I and II were mixed together as the PCR template to amplify Fny2bNLS and Fny2b6His fragments using the primer pair C2F1856/CMV123R. The resultant fragments were digested with HindIII and PstI and cloned into pFny209, generating the plasmids pFny209-2bNLS and pF209-2b6His. The sequences of primers C2F1856 and CMV123R have been documented elsewhere (34).

To express the 2b protein and its derivatives in Escherichia coli, coding sequences for Fny2b, Fny2bNLS, and Fny2b6His were amplified using pFny209 as the template with primer pairs F2b-BamHI/F2b-XhoI, F2b-BamHI/F2bNLS-XhoI, and F2b-BamHI/F2b6His-XhoI, respectively. The resultant PCR products were digested with BamHI and XhoI and cloned into pGEX-4t-1, generating the plasmids pGEX-4t-Fny2b, pGEX-4t-Fny2bNLS, and pGEX-4t-Fny2b6His. All constructs made in this work were authenticated by DNA sequencing. All primers used for generation of the DNA constructs described above are shown in Table 1.

**TABLE 1:** Primers used for generation of DNA constructs

Primer name	Primer sequence (5′–3′)
35S-XhoI	CATTTGGAGAGGACCTCGAG
GFP-R-SacI	TTGAGCTCACTTGTACAGCTCGTCCATG
F2b-E35A-F	GAAGGTCTCACAAACAGAATCGACGGGCACGAG
F2b-Q24R-R	CGATTCTGTTTGTGAGACCTTCGTCTCCGCTTC
F2b-K23R-R	CGATTCTGTTTGTGAGACCTTCGTCTCTGCCTCTTC
F2b-K23R-Q24R	CGATTCTGTTTGTGAGACCTTCGTCTCCGCCTCTTC
SacI-F2b-R	AAGAGCTCAGAAAGCACCTTCCG
F2b-NLS-SacI-R	AAGAGCTCATCGCCTGCGGCGCCTCTTGAAAGCACCTTCCGCCCA
F2b-6His-Sac-R	ATGAGCTCTCAGTGATGATGGTGATGATGTCCGAAAGCACCTTCCGCCCA
F2b-NLS-F	AAGAGGCGCCGCAGGCGATGAAACCTCCCCTTCCGC
F2b-NLS-R	GTTTCATCGCCTGCGGCGCCTCTTGAAAGCACCTTCCGCCCA
F2b-6His-F	GGACATCATCACCATCATCACTGAAACCTCCCCTTCCGC
F2b-6His-R	GTTTCAGTGATGATGGTGATGATGTCCGAAAGCACCTTCCGCCCA
F2b-BamHI	TTGGATCCATGGAATTGAACGTAGGTG
F2b-XhoI	TTCTCGAGTCAGAAAGCACCTTCCGCCCA
F2bNLS-XhoI	TTCTCGAGTCATCGCCTGCGGCGCCTCTTGAAAGCACCTTCCGCCCA
F2b6His-XhoI	TTCTCGAGTCAGTGATGATGGTGATGATGGAAAGCACCTTCCGCCCA

### Viruses and virus inoculation.

Generation of the mutant Fny-CMVΔ2bpro from Fny-CMV has been described previously (34). CMV mutants Fny-CMV2bNLS and Fny-CMV2b6His were constituted by coinoculating N. glutinosa plants with *in vitro*-synthesized transcripts of the infectious clones pFny209-2bNLS or pFny209-2b6His together with transcripts generated from pFny109 and pFny309. The genetic stability of these mutants was confirmed by sequencing reverse transcription (RT)-PCR products of RNA extracted from the inoculated plants. These viruses were passaged from the infected N. glutinosa to N. tabacum plants for virus propagation and virus purification. Virion purification was carried out as described by Ng and Perry (35). Purified virions at a concentration of 100 ng/μl were rub inoculated onto Arabidopsis seedlings at the 5- or 6-true-leaf stage using Carborundum as an abrasive. Successful infection was confirmed by symptom observation or by detection of CMV coat protein (CP) using a double-sandwich enzyme-linked immunosorbent assay (ELISA).

### Arabidopsis transformation.

A. tumefaciens cells carrying plasmid pBI121-EGFP, pBI121-EGFP-Fny2b, pBI121-EGFP-Fny2bNLS, or pBI121-EGFP-Fny2b6His were transformed into Arabidopsis ecotype Col-0 by floral dipping (36). Transformed Arabidopsis plants were selected on kanamycin-containing solid Murashige and Skoog medium and checked by observation of GFP fluorescence from leaf epidermal cells using a Leica SP5 confocal laser scanning microscope.

### Agroinfiltration assays.

For analysis of subcellular distribution of EGFP-Fny2b and its variants, A. tumefaciens cells harboring pBI121-EGFP or pBI121-EGFP-Fny2b or its variants were separately infiltrated into the fifth or sixth true leaves of N. benthamiana. For infiltration of A. tumefaciens, cells were diluted to a density equivalent to an *A*_600_ of 0.25. At 5 days postinfiltration, epidermal cells were imaged for GFP fluorescence by confocal microscopy. GFP fluorescence intensity in confocal images was quantified using the software LAS AF Lite (Leica).

For testing RNA silencing suppressor activities of Fny2b and its variants, A. tumefaciens cells carrying p35S:GFP were mixed with an equal amount of cells carrying pBI121-EGFP-Fny2b, pBI121-EGFP-Fny2bQ24R, pBI121-EGFP-Fny2bNLS, pBI121-EGFP-Fny2b6His, or control plasmids pBI121-GUS (β-glucuronidase) or pBI121-EGFP as described previously (37). A. tumefaciens (at final cell densities equivalent to an *A*_600_ of 0.5) were infiltrated into the fifth and sixth true leaves of N. benthamiana plants. GFP fluorescence in the infiltrated leaves was recorded under a UV lamp using a Nikon Coolpix digital camera.

### RNA blotting.

Total RNA was extracted from leaf tissues using TRIzol reagent (Invitrogen) according to the manufacturer's instructions. Northern blotting for analyses of CMV genomic RNAs and *GFP* mRNA were carried out according to a procedure described previously (37). The DNA oligonucleotide ProbeI-40 described previously (34) was labeled with biotin at its 3′ end for probing CMV genomic RNAs. The biotin-labeled probe was detected using a biotin-labeling detection kit (Beyotime, China). *GFP* mRNA was detected using a digoxigenin-labeled *GFP* probe, which was made using the digoxigenin (DIG) high primer DNA labeling and detection starter kit II (Roche) according to the manufacturer's instructions. For analysis of low-molecular-weight sRNAs, 15 μg of total RNA was used for Northern blot analysis according to the protocol described in the instructions of the miRVana miRNA isolation kit (Ambion). Mature miRNA or its star strand was detected using a complete cDNA oligonucleotide, which was labeled with digoxigenin at its 3′ ends using the DIG oligonucleotide tailing kit generation II (Roche), and purified using a G25 Sephadex column (GE). CMV siRNAs were detected using a battery of DIG-labeled DNA oligonucleotides corresponding to Fny-CMV passenger RNA3 (nucleotides 241 to 280, 741 to 780, 1341 to 1380, 1452 to 1491, 1499 to 1538, 1600 to 1639, 1681 to 1720, and 1731 to 1770). DIG-labeled DNA probes were detected using a chemiluminescence-based DIG detection kit (Roche) according to the manufacturer's instructions. U6 RNA was used as a loading control, and the sequence of the U6 probe has been described previously (38).

### RT-PCR.

Total RNA was isolated from aerial parts of Arabidopsis plants using TRIzol reagent and digested using Turbo DNase (Ambion). For analysis of relative accumulation of specific transcripts, RT-PCR quantitative assays were performed using a protocol previously described (39). The primers for Arabidopsis transcripts *PHABULOSA* (*PHB*), *AUXIN RESPONSE FACTOR* (*ARF*) *8*, and *AGO1* have been described previously (40, 41). *ELONGATION FACTOR 1A* (*EF1α*) was used as a reference transcript (42).

### Expression and purification of 2b proteins.

GST and GST-tagged 2b proteins were expressed from the pGEX-4t-1 vector in E. coli strain BL21. When bacterial cells had grown to a cell density corresponding to an *A*_600_ of approximately 0.6, 0.4 mM IPTG (isopropyl-β-d-thiogalactopyranoside) was added to induce protein expression, and the cells were incubated at 20°C for a further 4 h. Bacterial cells were harvested and sonicated on ice. Subsequently, proteins were purified using glutathione HiCap matrix slurry (Qiagen) according to the manufacturer's instructions. Just prior to carrying out RNA binding assays, purified proteins were passed through Amicon 10K columns (Millipore) for buffer exchange.

### 2b-RNA binding and EMSA.

For 2b-RNA binding and electrophoretic mobility shift assay (EMSA), three 21-nucleotide (nt) sRNAs based on the sequence of Arabidopsis miR168 were synthesized by TaKaRa. These synthetic RNAs were sRNA-1, which had a sequence identical to that of miR168 and was labeled at its 5′ end with biotin, sRNA-2, which had a sequence identical to that of miR168 star, and sRNA-3, which had 19 nucleotides completely complementary to sRNA-1. sRNA-1 was annealed to sRNA-2 or sRNA-3 in 10 mM Tris-HCl (pH 7.5), 1 mM EDTA, and 100 mM NaCl to form either ds-miRNA or ds-siRNA, respectively. Protein-RNA binding was performed according to the protocol previously described by González et al. (20). One microgram of GST or GST-tagged protein was incubated with 5 pmol ds-miRNA or ds-siRNA in binding buffer (20 mM Tris-HCl [pH 7.5], 1 mM dithiothreitol, 3 mM MgCl_2_, 50 mM NaCl) for 15 min on ice, resolved on 8% native PAGE gels, and transferred onto a nylon membrane (GE). Biotin-labeled sRNA was detected using a biotin-labeling detection kit (Beyotime, China).

### Immunoblotting.

For detection of free GFP or GFP-2b fusion proteins, total protein was extracted from leaf tissue, pulverized in liquid nitrogen, and homogenized using phosphate-buffered saline (0.14 M NaCl, 0.01 M potassium phosphate, pH 7.4) supplemented with 2% (vol/vol) 2-mercaptoethanol. Extracts were mixed with an equal volume of 2× Laemmli denaturation buffer (43) and boiled for 10 min. Protein extracts were separated by electrophoresis on SDS-containing 15% polyacrylamide gels (43) and transferred electrophoretically to nitrocellulose membranes (Whatman). Protein transfer and lane loadings were checked by Ponceau S staining. For detection of CMV CP, total soluble protein was extracted as previously described (44). Membranes were probed using polyclonal anti-GFP (Santa Cruz), anti-2b (19), or “home-made” anti-CP serum (44), and primary antibody binding was detected using horseradish peroxidase-conjugated anti-rabbit or anti-mouse IgG (Santa Cruz) and a chemiluminescence reagent kit (Thermo-Fisher) as previously described (44, 45).

## RESULTS

### Differential patterns of subcellular distribution of the 2b proteins from CMV strains of subgroups IA and II.

**FIG 1:**
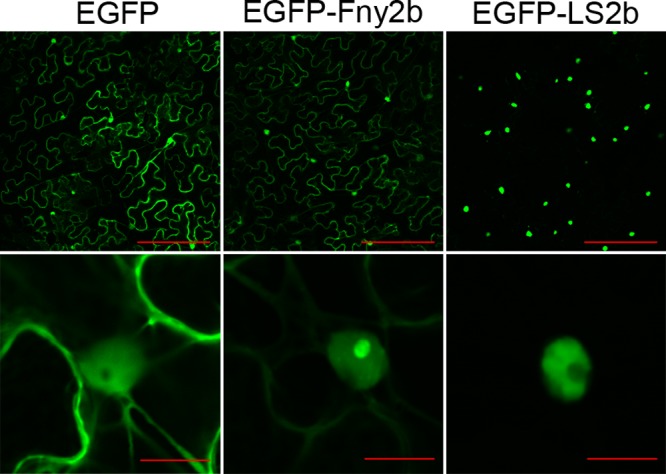
Subcellular distributions of the 2b proteins from subgroup IA strain Fny-CMV and subgroup II strain LS-CMV. Leaf epidermal cells of Nicotiana benthamiana in tissue agroinfiltrated to transiently express free EGFP, EGFP-Fny2b, or EGFP-LS2b were imaged by confocal scanning laser microscopy at 5 days postinfiltration. Bars, 200 μm (upper panels) and 10 μm (lower panels at higher magnification).

Previous studies suggested that the 2b proteins from subgroup II strains have stronger nuclear enrichment than 2b proteins of subgroup IA strains (18, 19, 28, 29). We ascertained the subcellular distributions of the 2b proteins from subgroup IA strain Fny-CMV and subgroup II strain LS-CMV by imaging plant tissues transiently expressing EGFP-2b fusion proteins following agroinfiltration. Leaf epidermal cells of N. benthamiana transiently expressing EGFP, EGFP-Fny2b, or EGFP-LS2b were imaged for GFP fluorescence by confocal scanning laser microscopy (Fig. 1). EGFP-Fny2b not only was found in nuclei but also was very apparent in the cytoplasm (Fig. 1). The subcellular distribution pattern appeared similar to that of free EGFP, except that EGFP-Fny2b, but not free EGFP, accumulated in nucleoli observed at higher magnifications (Fig. 1). EGFP-LS2b accumulated predominantly in nuclei, but with a limited amount appearing in the cytoplasm (Fig. 1). Interestingly, at higher magnifications it was apparent that EGFP-LS2b accumulated much less in the nucleolus than in the nucleoplasm (Fig. 1). Thus, there are marked differences in subcellular and subnuclear distribution between 2b proteins of subgroups IA and II CMV strains.

### Attachment of the NLS from LS2b to Fny2b dramatically enhanced nuclear targeting.

**FIG 2:**
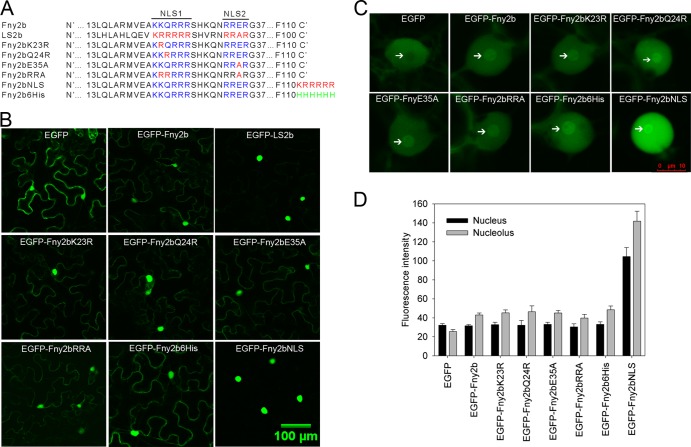
Subcellular distributions of Fny2b and its variants. (A) Schematic diagram of LS2b, Fny2b, and Fny2b-derivative proteins. The two NLS sequences (NLS1 and NLS2) are indicated in blue for Fny2b and in red for LS2b. Mutations to the NLS sequences in Fny2b are shown in red. Attachment of an NLS sequence (KRRRRR) or six histidine residues to the carboxyl end of Fny2b created the variant Fny2bNLS or Fny2b6His, respectively. (B to D) Transient expression of EGFP fusion proteins of Fny2b and its variants in N. benthamiana by agroinfiltration. Epidermal cells in infiltrated leaves were imaged at 5 days postinfiltration by confocal scanning laser microscopy. (B) Cytoplasmic and nuclear localization. (C) Magnified views of nuclei with nucleoli indicated by arrows. (D) Quantification of GFP fluorescence in nuclei and nucleoli as the mean value for 30 cells for each protein. Fluorescence intensities in nuclei and nucleoli were measured using the software LAS AF Lite (Leica). Bars represent standard errors of the means.

Nuclear localization of the 2b protein is determined by either one (subgroup II CMV strains) or two (subgroup IA) lysine- and arginine-rich NLSs (19, 28, 29). The single NLS from subgroup II LS 2b protein differs from NLS1 of subgroup IA Fny 2b protein by two residues (positions 23 and 24), and the corresponding sequence of LS2b differs from the Fny2b NLS2 sequence by one residue (position 35) (Fig. 2A). To determine if the LS2b NLS sequence is a stronger determinant of nuclear accumulation than the NLS of Fny2b and to understand the basis of the greater enrichment of LS2b in nuclei, we mutated either NLS1 or NLS2 or both in Fny2b to generate Fny2b mutants harboring LS-type NLS sequences. Respectively, these mutants were Fny2bK23R, Fny2bQ24R, Fny2bE35A, and Fny2bRRA (Fig. 2A). Subcellular distributions of these mutants were visualized by confocal microscope imaging of GFP fluorescence in leaf epidermal cells transiently expressing EGFP fusion proteins corresponding to each mutant. Significantly, all these mutants displayed accumulation patterns in the cytoplasm similar to those of Fny2b (Fig. 2B). We did not observe an alteration of nucleolar localization when these mutations were introduced into Fny2b (Fig. 2C). To further analyze the effects of these mutations on accumulation of EGFP-Fny2b in the nucleus and nucleolus, we measured fluorescence intensities of nuclei and nucleoli from 30 cells expressing each construct. Quantification data showed that fluorescent intensities of these mutants in the nucleus and nucleolus were equivalent to those from EGFP-Fny2b (Fig. 2D). These results demonstrated that placement of the NLS sequences of LS2b in the corresponding context in Fny2b was not sufficient to endow Fny2b with stronger, LS2b-like nuclear localization and that the nuclear-nucleolar-cytoplasmic partitioning properties of the two proteins are not defined solely by the characteristics of their respective NLS.

The NLS (_22_KRRRRR_27_) of the 2b protein from subgroup II strain Q-CMV efficiently directs a cytoplasmic protein into the nucleus when it is fused to the latter (29). To exaggerate the degree of nuclear localization of Fny2b, we fused its C terminus with a sequence identical to the NLS of LS2b to generate EGFP-Fny2bNLS. As a control, we added six histidine residues to the Fny2b protein sequence to create EGFP-Fny2b6His (Fig. 2A). EGFP-Fny2bNLS and EGFP-Fny2b6His were transiently expressed in N. benthamiana by agroinfiltration, and subcellular distribution was imaged by confocal microscopy at 5 days postinfiltration. Observation of green fluorescence showed that the control protein EGFP-Fny2b6His accumulated in both nucleus and cytoplasm, with a pattern that appeared indistinguishable from that of EGFP-Fny2b. However, EGFP-Fny2bNLS was detected only in nuclei (Fig. 2B), indicating that the attachment of the NLS sequence efficiently transports EGFP-Fny2b into the nucleus, sustains its accumulation there, and inhibits its accumulation in the cytoplasm. More highly magnified views of nuclei showed that both EGFP-Fny2b6His and EGFP-Fny2bNLS targeted nucleoli (Fig. 2C), indicating that the attachment of six histidine residues or the NLS sequence did not alter Fny2b nucleolar localization. Quantitative analysis of fluorescence intensity showed that EGFP-Fny2b6His and EGFP-Fny2b accumulated to a similar level in nuclei and nucleoli but that EGFP-Fny2bNLS accumulated to a much higher level in nuclei and nucleoli than either EGFP-Fny2b or EGFP-Fny2b6His (Fig. 2D).

### Forcing translocation of Fny2b into nuclei markedly impaired RNA-silencing suppressor activity.

**FIG 3:**
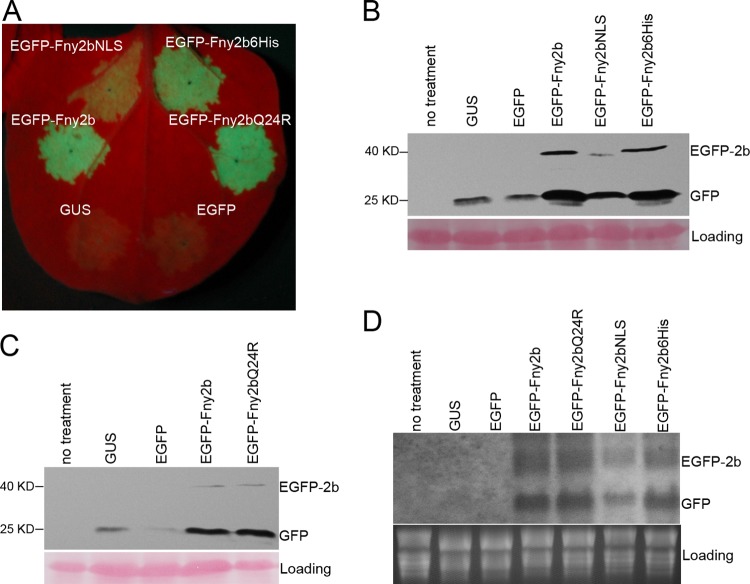
Effects of Fny2b and its variants on suppression of local RNA silencing. The binary vector pBI121-derived plasmid p35S:GFP expressing the reporter gene modified green fluorescence protein (*mgfp5*) was agroinfiltrated together with binary vectors expressing EGFP-Fny2b, its variants, or control proteins GUS and EGFP as indicated. (A) Infiltration patches photographed under UV light at 5 days postinfiltration. (B and C) Immunoblot analyses of accumulation of GFP and EGFP-fused 2b fusion proteins in agroinfiltrated patches at 5 days postinfiltration. A polyclonal anti-GFP serum was used to detect GFP and EGFP-fused 2b proteins. Ponceau S staining was used to monitor the equivalence of protein loading and transfer. (D) RNA gel blot analyses of steady-state accumulation of *GFP* and *EGFP-2b* transcripts in the agroinfiltrated patches. Total RNA was isolated from the agroinfiltrated patches at 5 days postinfiltration. *GFP* and *EGFP-2b* transcripts were detected using a digoxigenin-labeled GFP probe, which was made using the DIG high primer DNA labeling system with full-length *GFP* cDNA as the template. Equal loading was confirmed by staining of rRNA with ethidium bromide.

Studies using CMV2b NLS mutants had suggested that nuclear enrichment is required for CMV 2b to suppress siRNA-mediated RNA silencing and mediate CMV pathogenicity (11, 28, 29). More recently, however, González and colleagues (20) found that nuclear localization seemed to be dispensable for CMV 2b to inhibit siRNA-mediated RNA silencing in patch assays. To further explore the relative significance of cytoplasmic versus nuclear localization for CMV 2b VSR activity, we used agroinfiltration patch assays in N. benthamiana to test the abilities of EGFP-Fny2b, EGFP-Fny2bNLS, EGFP-Fny2b6His, EGFP-Fny2bQ24R, and control constructs expressing GUS and EGFP to inhibit local silencing of the modified GFP (mgfp5) as a reporter. At 5 days postinfiltration, leaf patches coinfiltrated with A. tumefaciens expressing either GUS or EGFP plus GFP exhibited extremely weak green fluorescence; however, leaf patches coexpressing EGFP-Fny2b and GFP showed strong green fluorescence, indicating that EGFP-Fny2b efficiently inhibited local silencing of GFP transcripts (Fig. 3A). Patches coinfiltrated with EGFP-Fny2bQ24R and GFP also exhibited strong fluorescence, which was comparable with that observed in the presence of EGFP-Fny2b (Fig. 3A). This was consistent with the equivalent accumulation of GFP protein and corresponding mRNA in the patches (Fig. 3C and D). Likewise, leaf patches coagroinfiltrated with EGFP-Fny2b6His and GFP exhibited equally strong fluorescence to that seen in tissue transiently expressing EGFP-Fny2b, demonstrating that the attachment of 6 histidine residues to the Fny2b C terminus did not affect 2b VSR activity. Interestingly, the patches coexpressing EGFP-Fny2bNLS and EGFP showed visible green fluorescence that, although substantially weaker than that found in the patches expressing EGFP-Fny2b or EGFP-Fny2b6His, was stronger than that seen in the patches expressing the control proteins GUS or EGFP (Fig. 3A). The difference in intensity of green fluorescence was supported by the analyses of steady-state accumulation of GFP protein using immunoblotting (Fig. 3B) and of *GFP* mRNA using RNA gel blotting (Fig. 3D). It was also supported by the relatively low levels of protein and mRNA of EGFP-Fny2bNLS compared with those of EGFP-Fny2b or EGFP-Fny2b6His (Fig. 3B and D). Taken together, all these data demonstrate that cytoplasmically localized 2b protein was a more efficient inhibitor of siRNA-mediated local RNA silencing than nucleus-localized 2b protein.

### Forced translocation of Fny2b into the nucleus diminished its ability to disrupt miRNA functions in transgenic Arabidopsis.

**FIG 4:**
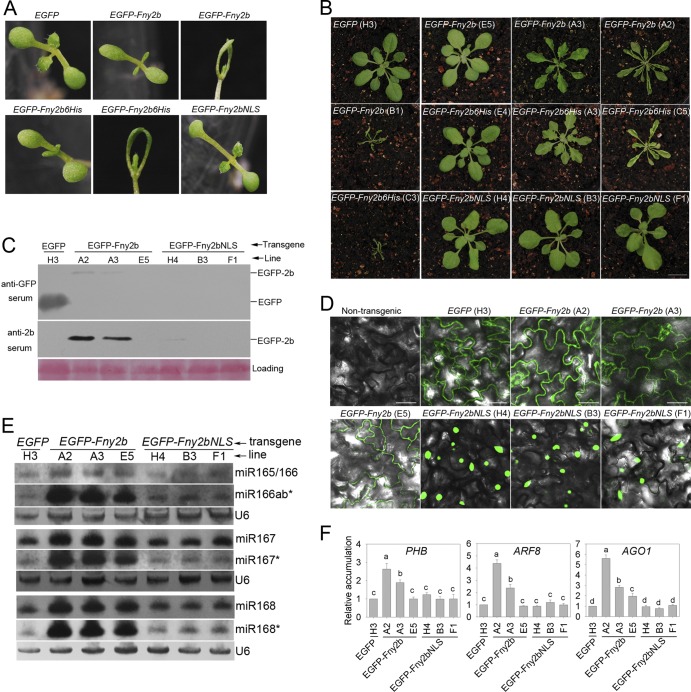
Translocation of Fny2b into the nucleus diminished its ability to disrupt microRNA functions in transgenic Arabidopsis. *EGFP*- or *EGFP-2b*-transgenic Arabidopsis plants were generated by transformation of binary vector pBI121-derived plasmids expressing free EGFP, EGFP-Fny2b, EGFP-Fny2bNLS (attachment of LS2b NLS to the C terminus of Fny2b), and EGFP-Fny2b6His (attachment of six histidine residues to the C terminus of Fny2b). (A) Typical phenotypes of *EGFP*- and *EGFP-2b*-transgenic lines at the cotyledon stage. *EGFP*-transgenic plants showed normal cotyledon morphology. *EGFP-Fny2b*-transgenic plants exhibited elongated and/or severely stunted cotyledons. *EGFP-Fny2b6His*-transgenic plants also exhibited elongated and severely unexpanded cotyledons. *EGFP-Fny2bNLS*-transgenic plants showed normal cotyledon development. (B) Typical phenotypes of *EGFP*- and *EGFP-2b*-transgenic lines in older plants (approximately 45 days after germination). The terms in parentheses indicate the line designations. (C) Detection of constitutive expression of EGFP and EGFP-fused 2b proteins in the transgenic lines using anti-GFP and anti-2b sera. (D) Confirmation of constitutive expression of EGFP or EGFP-2b proteins in the transgenic plants by observation of GFP fluorescence using a confocal microscope. Bar, 50 μm. (E) Northern blot analyses of accumulation of mature microRNAs and their star strands in transgenic plants as indicated. Total RNA was extracted from pooled aerial parts from five individual plants. U6 RNA was used as a loading control. (F) RT-qPCR analyses of accumulation in the transgenic lines of transcripts targeted by the microRNAs examined in panel E. *PHABULOSA* (*PHB*), *AUXIN RESPONSE FACTOR 8* (*ARF8*), and *ARGONAUTE 1* (*AGO1*) are targets of miR165/166, miR167, and miR168, respectively. *ARABIDOPSIS ELONGATION FACTOR 1α* (*EF1α*) was used as a reference transcript. The analysis of variance (ANOVA) Duncan's new multiple range test was used to analyze data for statistically significant differences. Different letters are assigned to results with statistically significantly different values (*P* < 0.05), while results assigned the same letter are not significantly different. Bars represent standard errors of the means.

Constitutive expression of Fny2b, but not Q2b or LS2b, disrupts miRNA functions in *2b*-transgenic Arabidopsis plants (23, 25, 26). To determine the relative significance of cytoplasmic and nuclear localization for Fny2b in its ability to interfere with miRNA activity, we generated lines of transgenic Arabidopsis plants, constitutively expressing EGFP, EGFP-Fny2b, EGFP-Fny2bNLS, or EGFP-Fny2b6His under the transcriptional control of the 35S promoter. In the T1 generation of *EGFP-Fny2b* transgenic plants, approximately 20% of the seedlings showed elongated cotyledons, and about 27% of the seedlings had very narrow, unexpanded cotyledons (Fig. 4A). Similarly, in the *EGFP-Fny2b6His*-transgenic T1 generation, approximately 25% and 23% of the seedlings presented elongated cotyledons or unexpanded cotyledons, respectively (Fig. 4A). To our surprise, none of the *EGFP-Fny2bNLS*-transgenic lines showed alteration of cotyledon development (Fig. 4A), which was identical to that of *EGFP*-transgenic lines. Typical phenotypes of aerial parts of adult (approximately 45 days after germination) plants of the transgenic lines are shown in Fig. 4B. None of the *EGFP*-transgenic lines showed developmental defects as exemplified by line H3 (Fig. 4B). Of 53 *EGFP-Fny2b*-transgenic lines, 24 lines showed no obvious changes to phenotypes as exemplified by line E5 and the other 29 lines showed phenotypic alterations to a varied extent (Fig. 4B). Of those 29 lines, three lines with mild defects were represented by line A3, showing uncurled or slightly curled rosette leaves with strong serration. Twenty-five lines were represented by line A2, exhibiting narrow, serrated, and strongly upwardly curled leaves. One line, line B1, showed a highly altered phenotype of extremely severe effects on growth and development; these plants were tiny with narrow leaves growing upwards. The *EGFP-Fny2b6His*-transgenic lines showed patterns similar to those of the *EGFP-Fny2b*-transgenic lines in terms of altered phenotypes and proportions of T1 lines showing developmental defects (Fig. 4B). However, the *EGFP-Fny2bNLS*-transgenic lines displayed distinctly different phenotypic changes from those displayed by the lines expressing either EGFP-Fny2b or EGFP-Fny2b6His. Of 59 *EGFP-Fny2bNLS*-transgenic lines, 24 lines showed no developmental defects as exemplified by the line B3, 27 lines showed mild down-curled rosette leaves represented by line H4, and 8 lines showed scattered necrosis on rosette leaves represented by line F1 (Fig. 4B). These results demonstrated that constitutive expression of EGFP-Fny2bNLS had much less effect on plant development than expression of EGFP-Fny2b or EGFP-Fny2b6His.

Expression of EGFP, EGFP-Fny2b, and EGFP-Fny2bNLS in their respective transgenic lines was analyzed by using immunoblots with sera against GFP or 2b and by visualizing GFP fluorescence in leaf epidermal cells. Immunoblot analysis using an anti-GFP serum showed that GFP was detectable in the *EGFP*-transgenic line, and EGFP-Fny2b was detected in its transgenic lines A2 and A3, but not in line E5. Line A2 displayed a slightly higher level of EGFP-Fny2b than line A3 (Fig. 4C). We observed a similar expression pattern for EGFP-Fny2b in these three lines using an anti-2b serum (Fig. 4C). The different expression levels of EGFP-Fny2b correlated with the severity of developmental defects in these lines (Fig. 4B), which was consistent with the CMV *2b*-transgenic plants previously reported (23, 30). However, we detected expression of EGFP-Fny2bNLS only in its transgenic line H4 using the anti-2b serum (Fig. 4C). Strikingly, we observed GFP fluorescence in all these transgenic lines (Fig. 4D). Consistent with the immunoblot result, GFP fluorescence in the *EGFP-Fny2b*-transgenic line E5 was much weaker than that in lines A2 and A3. These three *EGFP-Fny2b*-transgenic lines exhibited GFP fluorescence that was predominantly located in the cytoplasm. In contrast, EGFP-Fny2bNLS was strongly enriched in the nuclei, which was consistent with the observations in N. benthamiana (Fig. 2B).

Phenotypic alterations in transgenic plants expressing viral suppressors are associated with disruption of miRNA-mediated regulation of host target mRNAs (23, 25, 26, 46, 47, 48). First, we compared the accumulation levels of four miRNA families in the *EGFP*-, *EGFP-Fny2b*-, and *EGFP-Fny2bNLS*-transgenic lines as shown in Fig. 4E. These miRNAs were miR165, miR166, miR167, and miR168, which are upregulated by the expression of Fny2b in transgenic Arabidopsis plants (23). RNA samples used for RNA gel blotting were isolated from aerial parts of transgenic plants from the T2 generation. RNA gel blot analyses showed that accumulation of all those mature miRNAs and their star miRNAs increased in all three *EGFP-Fny2b*-transgenic lines (Fig. 4E). However, accumulation of these miRNAs showed no obvious changes in the *EGFP-Fny2bNLS*-transgenic lines compared with those in the *EGFP*-transgenic line (Fig. 4E).

Next, we analyzed steady-state accumulation of the target transcripts for the four miRNAs: *PHB* for miR165 and miR166, *ARF8* for miR167, and *AGO1* for miR168 using quantitative RT-PCR (RT-qPCR) (Fig. 4F). In the *EGFP-Fny2b*-transgenic lines A2 and A3, accumulation of all target transcripts increased. However, in the *EGFP-Fny2b*-transgenic line E5, only *AGO1* increased slightly in accumulation compared with that in the *EGFP*-transgenic line. The accumulation of these target transcripts correlated with the severity of phenotypic defects in the *EGFP-Fny2b*-transgenic lines. However, accumulation of target transcripts did not increase in the *EGFP-Fny2bNLS*-transgenic lines (Fig. 4F). Taken together, all these results demonstrate that exaggerating the translocation of Fny2b into the nucleus diminished the ability of Fny2b to disrupt miRNA functions in Arabidopsis.

### Attachment of an NLS sequence did not alter the ability of Fny2b to bind to small RNAs.

**FIG 5:**
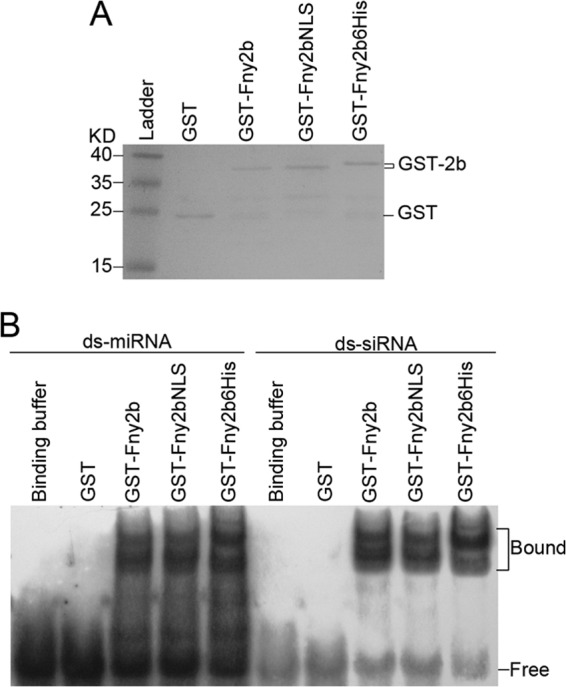
Modification of the Fny2b protein by addition of either an additional NLS or a hexahistidine sequence did not compromise binding to double-stranded small RNAs. (A) SDS-PAGE analysis of GST and GST-2b fusion proteins synthesized in E. coli and purified by affinity chromatography confirmed successful expression of GST-Fny2b, GST-Fny2bNLS, and GST-Fny2b6His. Protein bands were visualized by staining with Coomassie blue. (Ladder) indicates a lane loaded with protein molecular mass markers. (B) Electrophoretic mobility shift assays for assessing the abilities of 2b protein variants to bind miRNA and siRNA duplexes. Biotin-labeled miRNA and siRNA duplexes (ds-miRNA and ds-siRNA) were produced by annealing Arabidopsis miR168 sequence-based, biotin-labeled sRNA-1 with either miR168 star sequence-based sRNA-2 or with sRNA-3, respectively. ds-miRNA or ds-siRNA (5 pmol) was incubated with 1 μg purified GST or GST-tagged 2b proteins, resolved by 8% acrylamide native PAGE, and blotted onto a nylon membrane. Biotin-labeled RNAs were detected using horseradish peroxidase conjugated to streptavidin. The positions of bands corresponding to free and protein-bound (Bound) RNAs are indicated.

CMV 2b protein suppresses RNA silencing predominantly by sequestering sRNA duplexes to prevent their recruitment into the RNA-induced silencing complex (RISC) (18–21). As shown above, enhancing transport of Fny2b into nuclei diminished its silencing suppressor activity (Fig. 3 and 4). To rule out the possibility that compromised silencing suppressor activity resulted from decreased ability to bind sRNA, we conducted *in vitro* 2b-sRNA binding assays. Glutathione *S*-transferase (GST) or GST-tagged Fny2b, Fny2bNLS, and Fny2b6His were expressed and purified from E. coli (Fig. 5A) and incubated with either Arabidopsis miR168-based synthetic miRNA (ds-miRNA) or siRNA (ds-siRNA) duplexes, and the resulting protein-RNA interactions were detected by EMSA (Fig. 5B). EMSA showed that, as expected, GST did not bind ds-miRNA or ds-siRNA but that binding was exhibited by GST-tagged Fny2b (Fig. 5B). It was also found that Fny2bNLS and Fny2b6His exhibited a degree of affinity for the synthetic ds-miRNA and ds-siRNA identical to that of Fny2b (Fig. 5B). This demonstrated that the attachment of a hexahistidine or NLS sequence did not alter the ability of Fny2b to bind sRNAs. Thus, the compromised silencing suppressor activity of Fny2bNLS seen *in planta* resulted from the alteration in its subcellular localization and not from any change in its ability to sequester sRNAs.

### Exaggerating nuclear enrichment of Fny2b enhanced viral pathogenicity.

**FIG 6:**
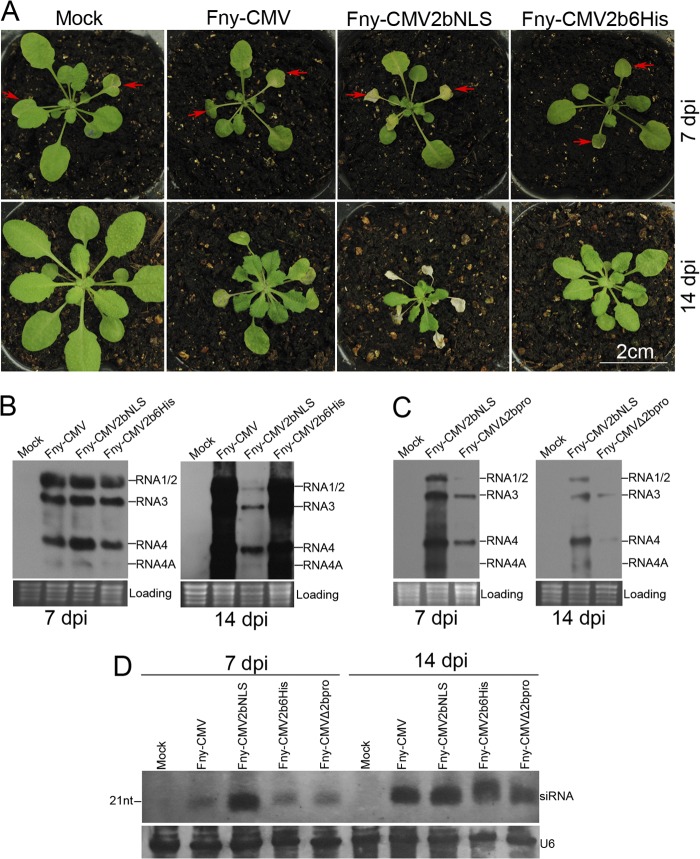
Increased translocation of Fny2b into the nucleus enhanced virus virulence but did not increase virus accumulation. (A) Viral symptoms in wild-type Arabidopsis infected with Fny-CMV, Fny-CMV2bNLS, and Fny-CMV2b6His. Water-inoculated plants are indicated (Mock). Arrowheads indicate inoculated leaves. Photographs were taken at 7 dpi and 14 dpi as indicated on the right. (B and C) Northern blot analyses of accumulation of viral progeny RNAs in the plants as shown in panel A and the plants infected with Fny-CMVΔ2bpro, which expresses no 2b protein at all. Total RNA was extracted from pooled aerial parts from five individual plants at 7 dpi and from pooled top leaves from five individual plants at 14 dpi. A biotin-labeled DNA oligonucleotide complementary to the highly conserved sequence of the 3′ untranscribed region (UTR) of the CMV genomic and subgenomic RNAs was used to detect viral RNA. Equal loading was confirmed by ethidium bromide staining of rRNA. (D) Northern blot analyses of accumulation of viral siRNAs. Total RNA was extracted from plant samples as described in panels B and C. A mixture of 8 DIG-labeled DNA oligonucleotides corresponding to CMV RNA3 was used to probe viral siRNAs. U6 RNA was used as a loading control.

As shown above, targeting Fny2b into nuclei compromised its ability to suppress not only siRNA- but also miRNA-mediated RNA silencing (Fig. 3 and 4). Thus, we speculated that enhancing nuclear accumulation of Fny2b would reduce viral accumulation and ameliorate viral symptoms during CMV infection. To test this, we generated CMV mutants Fny-CMV2bNLS and Fny-CMV2b6His, in which the 2b protein of Fny-CMV was appended at its C terminus with the LS2b NLS sequence (KRRRRR) and six histidine residues, respectively. It was expected that the extra NLS would have the same effect on the subcellular localization of 2b as on that of GFP-2b. We compared these mutants with wild-type Fny-CMV in terms of disease symptoms and virus titer in Arabidopsis ecotype Col-0. Observation of viral symptoms showed that infection with Fny-CMV2b6His caused slightly less severe disease symptoms than Fny-CMV (Fig. 6A). However, RNA gel blot analysis indicated that there was no obvious difference in virus RNA accumulation between the wild-type CMV and Fny-CMV2b6His at 7 or 14 days postinoculation (dpi) (Fig. 6B). Thus, the attachment of six histidine residues did not affect virus titer but did slightly reduce viral pathogenicity. Unexpectedly, infection with Fny-CMV2bNLS induced more-severe symptoms than Fny-CMV. Leaves inoculated with Fny-CMV2bNLS began to exhibit necrosis at about 3 dpi (data not shown) and had died by 7 dpi (Fig. 6A). This is in marked contrast to the effects of Fny-CMV and Fny-CMV2b6His, neither of which induced necrosis (Fig. 6A). Necrosis was also observed at approximately 7 dpi in those upper, systemically infected leaves that had already developed at the time of plant inoculation with Fny-CMV2bNLS, and these leaves had died by 14 dpi (Fig. 6A). However, in these plants, the leaves that developed after Fny-CMV2bNLS inoculation showed only distortion but no necrosis. We noted that Fny-CMV2bNLS caused viral symptoms of distortion in the topmost leaves approximately 1 day earlier than Fny-CMV or Fny-CMV2b6His (data not shown). RNA gel blot analyses showed that Fny-CMV2bNLS accumulated at a level similar to that of Fny-CMV or Fny-CMV2b6His in whole aerial parts of plants at 7 dpi but at a dramatically lower level than the wild-type virus and Fny-CMV2b6His in the top leaves by 14 dpi (Fig. 6B). Both of these mutant viruses were genetically stable, which was confirmed by sequencing of cDNA synthesized from RNA extracted from systemically infected leaves using RNA-specific primers. The results demonstrated that increasing targeting of the Fny2b protein into the nucleus enhanced CMV pathogenicity, although it did not cause a sustained increase in virus titer in Arabidopsis.

To ascertain how effective nucleus-localized Fny2bNLS was in suppression of antiviral RNA silencing, we compared virus titers in Arabidopsis plants infected with Fny-CMV2bNLS and with mutant Fny-CMVΔ2bpro, which expresses no 2b protein at all (34). Northern blot analyses showed that accumulation levels of Fny-CMV2bNLS RNA were markedly higher than those of Fny-CMVΔ2bpro RNA at 7 dpi and slightly higher than those of Fny-CMVΔ2bpro RNA at 14 dpi (Fig. 6C). This strongly suggests that nucleus-localized 2b protein possesses at least some ability to inhibit antiviral RNA silencing but that by itself this activity is not sufficient to prevent silencing-mediated inhibition of virus accumulation in the newly developed leaves. To assess the relationship between viral RNA accumulation and the buildup of siRNAs during infection, we analyzed accumulation of virus-derived siRNAs in upper, systemically infected leaves at 7 dpi and 14 dpi. At 7 dpi, although plants infected with Fny-CMV2bNLS, Fny-CMV, and Fny-CMV2b6His contained similar levels of viral RNA (Fig. 6B), the levels of virus-derived siRNAs in plants infected with Fny-CMV2bNLS were markedly higher than those in plants infected with Fny-CMV, Fny-CMV2b6His, or, remarkably, Fny-CMVΔ2bpro, which is unable to produce a VSR (Fig. 6D). This demonstrated that Fny2bNLS was not as efficient as Fny2b or Fny2b6His in suppressing silencing, as indicated by a more rapid buildup of virus-derived siRNAs by 7 dpi. By 14 dpi, virus-derived siRNAs had reached similar levels in plants infected with Fny-CMV, Fny-CMV2b6His, Fny-CMVΔ2bpro, or Fny-CMV2bNLS (Fig. 6D). Overall, no relationship was observed between accumulation levels of viral genomic RNAs and siRNAs except in the case of plants infected with Fny-CMV2bNLS, in which accumulation of virus-derived siRNA seemed to occur earlier. Thus, the rapidity with which virus-derived siRNAs accumulate, rather than their eventual steady-state level, is more important in determining the eventual titer of CMV.

### Weak VSR activity of nucleus-localized 2b protein is responsible for decreased virus titer.

**FIG 7:**
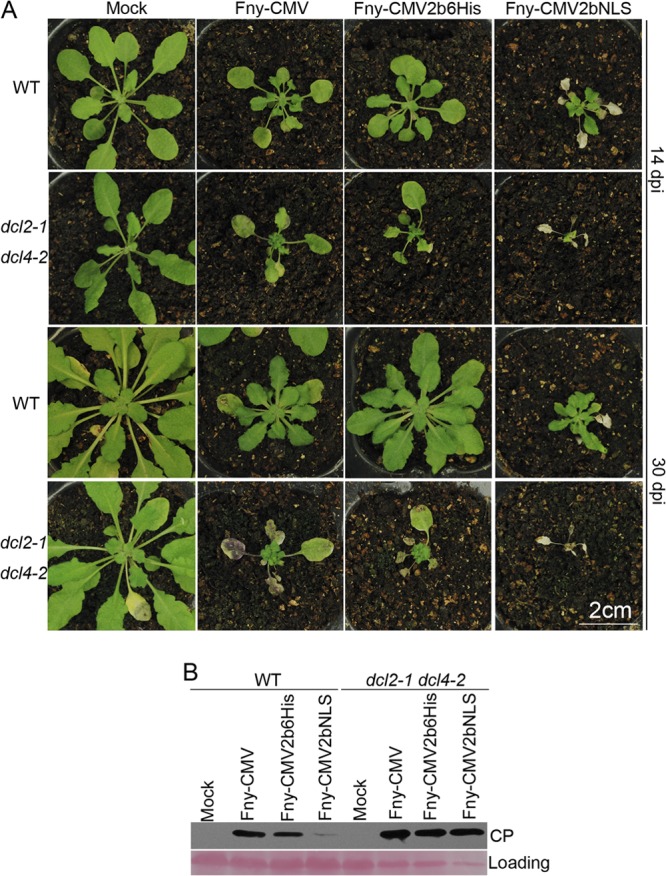
Fny-CMV2bNLS was rescued in Arabidopsis *dcl2-1*/*dcl4-2* double mutant plants and killed the mutant hosts. (A) Disease symptoms on *A. thaliana* wild-type Col-0 (WT) and *dcl2-1*/*dcl4-2* double mutant plants inoculated with Fny-CMV, Fny-CMV2b6His, Fny-CMV2bNLS, or water (Mock). Photographs were taken at 14 dpi and 30 dpi as indicated on the right. (B) Immunoblot analysis of CMV CP accumulation in the upper, still-living leaves at 14 dpi. CP was detected using a polyclonal serum against CMV CP. Ponceau S staining was used to monitor the equivalence of protein loading and transfer.

As shown above, exaggeration of the partitioning of Fny2b into the nucleus compromised 2b VSR activity in the patch assay (Fig. 3) and resulted in decreased CMV RNA accumulation in the recovered tissues of infected Arabidopsis at 14 dpi (Fig. 6B). To determine definitively whether the compromised VSR activity is responsible for the decreased CMV RNA titer, we examined the effects of Fny-CMV, Fny-CMV2b6His, and Fny-CMV2bNLS on *dcl2-1/dcl4-2* double mutant plants, in which siRNA-mediated resistance to viruses is largely abolished (30). Compared with wild-type plants, *dcl2-1/dcl4-2* double mutant plants showed more-severe disease symptoms in response to Fny-CMV and both of the mutants (Fig. 7A). As expected, by 14 dpi, infection with Fny-CMV2bNLS had caused necrosis in the mature, noninoculated leaves of wild-type plants. However, in *dcl2-1/dcl4-2* double mutant plants the necrosis induced by Fny-CMV2bNLS was far more widespread and also encompassed newly emerged upper leaves (Fig. 7A). Even at 30 dpi, *dcl2-1/dcl4-2* double mutant plants infected with either Fny-CMV or Fny-CMV2b6His were still alive, but those infected with Fny-CMV2bNLS were dead (Fig. 7A). It was noted that the difference in viral symptoms between Fny-CMV and Fny-CMV2b6His in wild-type plants was not apparent in *dcl2-1/dcl4-2* mutant plants. Next, we tested virus levels in the upper leaves at 14 dpi using immunoblot analysis for the CMV CP. In wild-type Arabidopsis, infection with Fny-CMV2bNLS resulted in a much lower level of CP than did infection with either Fny-CMV or Fny-CMV2b6His, and the last two showed a comparable CP level (Fig. 7B). This was consistent with the results from RNA gel blot analysis for viral RNAs (Fig. 6B). However, in *dcl2-1/dcl4-2* double mutant plants infected with Fny-CMV2bNLS, CP accumulated to a level comparable to that in plants with Fny-CMV or Fny-CMV2b6His (Fig. 7B). Taken together, all these results demonstrate that compromised VSR activity is responsible for the decreased virus titer, while suggesting that it is the exaggerated levels of 2b in the nucleus that induced systemic necrosis.

### Systemic necrosis caused by Fny-CMV2bNLS was independent of its effects on sRNA pathways.

**FIG 8:**
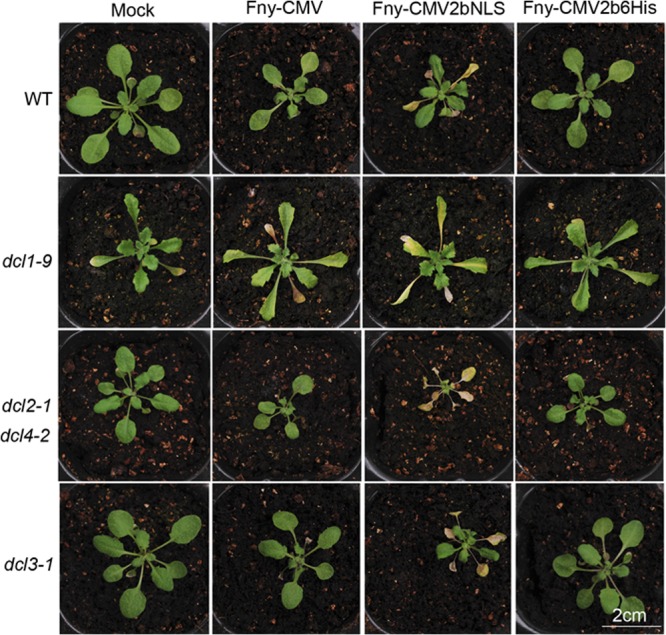
Systemic necrosis caused by infection with the mutant Fny-CMV2bNLS is unrelated to effects on small RNA pathways. Arabidopsis wild-type (WT) and mutants deficient in microRNA biogenesis (*dcl1-9*) or siRNA biogenesis (*dcl2-1/dcl4-2* and *dcl3-1*) were inoculated with Fny-CMV, Fny-CMV2bNLS, or Fny-CMV2b6His. Mock, plants were mock inoculated with sterile water. Plants were photographed at 14 dpi with the exception of *dcl1-9* mutant plants, which were imaged at 10 dpi.

Enhanced targeting of the Fny2b into the nucleus compromised its VSR activity (Fig. 3 and 4) but enhanced 2b-mediated CMV virulence (Fig. 6 and 7). To determine whether or not the enhanced virulence, manifested as systemic necrosis, was mediated by the effects of nucleus-localized 2b protein on miRNA and/or siRNA pathways, we examined viral symptoms in wild-type Arabidopsis plants and plants of the following mutant lines: *dcl1-9*, deficient in miRNA biogenesis; *dcl3-1*, deficient in 24-nt siRNA biogenesis, and the *dcl2-1/dcl4-2* double mutant (deficient in antiviral silencing). Plants were inoculated with Fny-CMV, Fny-CMV2bNLS, or Fny-CMV2b6His. All mutant lines infected with Fny-CMV or Fny-CMV2b6His displayed no necrosis in systemically infected leaves, but plants infected with Fny-CMV2bNLS presented systemic necrosis (Fig. 8). These results support the idea that the enhanced virulence was unrelated to effects of nucleus-localized 2b protein on miRNA- or siRNA-regulated target functions.

## DISCUSSION

We investigated the contributions of nucleus-localized 2b to RNA-silencing suppression and virulence. Our data support the contention that the VSR activity of CMV 2b protein is predominantly exerted by that portion of the 2b protein residing in the cytoplasm and that it is this population of 2b protein molecules that is required for maximal suppression of antiviral silencing (20, 49). However, we also showed that nucleus/nucleolus-localized 2b protein does possess VSR activity, albeit of insufficient strength to completely inhibit the occurrence of antiviral silencing in the youngest, still-developing host tissues. Moreover, nucleus/nucleolus-localized 2b protein is strongly associated with CMV virulence. We found that the NLS of LS2b significantly enhanced not only nuclear but also nucleolar enrichment of Fny2b when it was fused to the C terminus of GFP-Fny2b. Exaggerating the nuclear and nucleolar enrichment of Fny2b markedly compromised its VSR activity but enhanced CMV virulence, making the virus necrogenic and accelerating the appearance of disease symptoms in Arabidopsis plants.

### Subcellular distribution and nucleolar localization of CMV 2b proteins.

Our data demonstrate that there are marked differences in subcellular distribution between 2b proteins of subgroups IA and II CMV strains. The differential subcellular distribution can be attributed to the differences between the NLS sequences of 2b proteins from subgroups IA and II, based on the previous finding that cytoplasmic localization of GFP-Q2b increases when the third residue (arginine) of the NLS in Q2b is replaced with glutamine to simulate the sequence in Fny2b (29). Unexpectedly, we found that the LS2b NLS did not substantially alter subcellular distribution of Fny2b when it was located at the original context in Fny2b. In contrast, it strongly enhanced nuclear translocation of Fny2b when it was fused to the C terminus of Fny2b. This finding demonstrates that the LS2b NLS at the original context in Fny2b is not sufficient to transport Fny2b into the nucleus. The low similarity (48.5%) between the protein sequences of Fny2b and LS2b allows us to speculate that Fny2b has different local structures surrounding its NLS than does LS2b and that these local structures are critical for recognition of the Fny2b NLS1 sequence by specific host importin factors, probably by interaction with karyopherin α as suggested previously (28). Additionally, we found an interesting CMV strain-specific phenomenon in that LS2b was apparently absent in nucleoli, which is in contrast to Fny2b or SD2b (18, 19). While NLS1 and NLS2 of Fny2b and SD2b are needed for nucleolar localization (18, 19), our data demonstrated that nucleolar localization of Fny2b is not determined solely by the NLS domains. It should be considered that, besides NLS1 and NLS2 domains, other portions of Fny2b may be required for its nucleolar localization. This is consistent with the delineation of the 2b nucleolar localization signal to the N-terminal 13- to 37-amino-acid sequence domain (18), and indeed 10 residues differ between Fny2b and LS2b in this region (Fig. 2A). Although the NLS sequence of LS2b does not possess the ability to target LS2b into the nucleolus, fusing this NLS sequence to Fny2b enhanced not only the nuclear but also the nucleolar accumulation of Fny2b. This demonstrated that nuclear accumulation is important, but not sufficient, for CMV 2b to accumulate in nucleoli.

### Relationship of the subcellular distribution of CMV 2b protein with VSR activity and viral pathogenicity.

We found that increasing translocation of Fny2b into the nucleus and nucleolus greatly compromised its ability to inhibit siRNA-mediated local RNA silencing, antiviral silencing, and miRNA activity. This allowed us to conclude that suppression of sRNA pathways by CMV 2b takes place primarily in the cytoplasm, as suggested previously (20, 48). CMV 2b protein inhibits RNA silencing predominantly by sequestering sRNAs to prevent their entry into the RISC (18–21), but it also inhibits AGO1 (23). Similar to CMV 2b, other VSRs, including the potyviral HC-Pro and tombusviral P19, bind sRNAs (17, 50) and reside mainly in the cytoplasm (51, 52). Translocation of P19 into the nucleus greatly impairs its VSR activity (53), demonstrating that binding sRNAs by P19 occurs in the cytoplasm. Recently, AGO1 was identified as a peripheral membrane protein in the cytoplasm (54) and its interaction with Fny2b was observed mainly in the cytoplasm (19). Therefore, this element of 2b activity, at least, is likely to occur in this cellular compartment, although it should be remembered that AGO1 binding is not critical for VSR activity (18, 19).

Although the nucleus- and nucleolus-localized 2b protein variant EGFP-Fny2bNLS showed weak VSR activity in patch assays (Fig. 3), its analogue Fny2bNLS efficiently enhances virus accumulation early in infection, albeit with higher accumulation of viral siRNAs, but this was not sustained, leading to a reduced viral accumulation later in infection (Fig. 6). Thus, although nucleus and nucleolus-localized 2b protein has VSR activity, it is not strong enough to inhibit antiviral silencing. Our conclusion is consistent with previous findings that nuclearly localized Q2b is weaker than Fny2b and SD2b in silencing suppression in the agroinfiltration patch assays (29, 55) and that replacement of the 3′ terminus of Fny-CMV RNA2 with that of LS RNA2, which includes the region of the *2b* gene, decreases virus accumulation in tomato (56). However, in contrast, when constitutively expressed in transgenic plants, the Fny2b and LS2b proteins were equally effective in negating self-silencing of a GFP-expressing, potato virus X (PVX)-derived amplicon (26).

Previous transgenic plant experiments have demonstrated that the 2b proteins from subgroup IA strains Fny and SD, but not those from subgroup II strains LS and Q, disrupted miRNA functions in Arabidopsis (18, 23, 25, 26). The undetectability of Q2b protein in transgenic plants using immunoblot analysis was used to suggest that the 2b protein is unstable *in planta*, which is a possible explanation for the inability of Q2b or LS2b to disrupt miRNA functions (23). Here, we also found that constitutive expression of the nuclear/nucleolar EGFP-Fny2bNLS was barely detected in its transgenic plants using immunoblotting, but in fact, it stably accumulated in nuclei as shown by the observation of strong GFP fluorescence, and yet it had no effect on miRNA functions (Fig. 4). As stated above, Fny2bNLS displayed a pattern of subcellular distribution similar to that of LS2b or Q2b when fused with GFP. Thus, we speculate that strong partitioning into the nucleus, rather than instability, could be the reason for the failure of LS2b or Q2b to significantly disrupt miRNA activity in Arabidopsis.

Our data indicate that there is a relationship between CMV pathogenicity and 2b nuclear/nucleolar localization in Arabidopsis plants, since increasing the accumulation of Fny2b in the nucleus and nucleolus enhanced virulence, even to the extent of inducing necrosis. However, necrosis does not occur in Arabidopsis plants infected with the CMV subgroup II strain LS or Q, both of which express 2b proteins that are predominantly nuclearly localized (10, 42). Thus, the differences in subnuclear localization between Fny2bNLS (which accumulates in the nucleus and nucleolus) and LS2b (which accumulates in the nucleus but not in the nucleolus) suggest that enhanced nucleolar localization of Fny2b contributes in some way to the elicitation of severe symptoms, including necrosis. For the moment, the mechanism by which localization of the 2b protein to the nucleolar compartment affects virulence remains elusive. The nucleus/nucleolus-localized Fny2bNLS is a weak VSR *in planta*, and necrosis occurred in the various *dcl* mutants infected with Fny-CMV2bNLS, indicating that in this case, increased virulence and necrosis are not induced through interference with sRNA pathways. CMV2b has been shown to be a transcriptional activator in yeast (57) and binds DNA *in vitro* (58). We speculate that the distribution of 2b protein between the cytoplasmic, nuclear, and nucleolar compartments may contribute to CMV pathogenicity by misregulation of transcription of host genes controlling plant development or programmed cell death.
